# DPD Simulation on the Transformation and Stability of O/W and W/O Microemulsions

**DOI:** 10.3390/molecules27041361

**Published:** 2022-02-17

**Authors:** Menghua Li, Haixia Zhang, Zongxu Wu, Zhenxing Zhu, Xinlei Jia

**Affiliations:** 1 Department of Chemical Engineering and Safety, Binzhou University, Binzhou 256603, China; k_mengmeng@163.com; 2 Binzhou Dayou New Energy Development Company Limited, Binzhou 256600, China; chnchemwzx@163.com; 3 Binzhou City Building and Design Institute, Binzhou 256600, China; zzx1025286003@126.com

**Keywords:** microemulsion, interfacial tension, end to end distance, dissipative particle dynamics (DPD) simulation

## Abstract

The dissipative particle dynamics simulation method is adopted to investigate the microemulsion systems prepared with surfactant (H1T1), oil (O) and water (W), which are expressed by coarse-grained models. Two topologies of O/W and W/O microemulsions are simulated with various oil and water ratios. Inverse W/O microemulsion transform to O/W microemulsion by decreasing the ratio of oil-water from 3:1 to 1:3. The stability of O/W and W/O microemulsion is controlled by shear rate, inorganic salt and the temperature, and the corresponding results are analyzed by the translucent three-dimensional structure, the mean interfacial tension and end-to-end distance of H1T1. The results show that W/O microemulsion is more stable than O/W microemulsion to resist higher inorganic salt concentration, shear rate and temperature. This investigation provides a powerful tool to predict the structure and the stability of various microemulsion systems, which is of great importance to developing new multifunctional microemulsions for multiple applications.

## 1. Introduction

Microemulsion (ME) is a single optically isotropic and thermodynamically stable liquid solution with particle sizes of less than 100 nm and up to 200 nm, consisting of two immiscible liquids such as oil, water or an organic solvent [1]. One phase called the droplet or dispersed phase is embedded in another phase named the continuous phase [2]. According to the spread and continuous phase, microemulsion may be classified into the following three types: water in oil (W/O) that oil is the continuous phase, oil in water (O/W) that water is the continuous phase, and bicontinuous phase, as defined by Liu et al. and Yew et al. [3,4]. Microemulsions with the particular structure are of great importance in many industrial fields, such as the adsorption and the skin penetration of drugs [5,6,7,8,9], oil recovery [10,11], heck reactions [12], and luminescent solar concentrator [13] because of the particular characteristics of ultralow interfacial tension, sizeable interfacial area, high solubilization and low viscosity.

Surfactant is an essential component always used in microemulsion by their amphiphilic nature. A surfactant consisting of a hydrophobic hydrocarbon tail and a hydrophilic polar head group could decrease the interfacial tension and negatively interfere with the phase-separation process to obtain long-term stable emulsion [14,15,16]. Emulsions are thermodynamic unstable and prone to coalescence, sedimentation, flocculation, and other phenomena. The surfactant molecules exert their role as interface stabilizers, which could migrate toward the oil-water interface and inhibits coalescence [17]. In which, the part of surfactant tail length involves van der Waals interactions between their hydrocarbon chains. Posocco et al. observe the intermolecular hydrophobic interaction forces that are more robust with longer hydrophobic tails (at least eight carbon atoms), eventually, the corresponding interfacial film is more stable [2]. 

Previous studies about the affecting factors for emulsions including the composition of the emulsion, pH, ionic strength, shear and cryoprotectants on the stability have been carried out over the last years [17,18,19,20,21,22]. Okuro et al. observed the phase inversion of W/O high internal phase emulsions (HIPEs) to O/W emulsions with higher energy input. Moreover, the shear-thinning behavior and instability were obtained in all W/O-HIPEs at a high shear rate, whereas O/W emulsions showed greater viscosity and stability [23]. Huang et al. observed that the temperature, water–oil ratio and HLB value could influence the emulsion stability and emulsion form as O/W or inverse W/O. The inverse W/O emulsion was found to be the most stable with different affecting factors [24]. Zhong et al. showed that the addition of salt ions resulted in an increased extent of interfacial-protein adsorption and proved to be more durable. When 100 mM salt was added, the emulsions had the best stability [20]. Furthermore, the stability of fuel microemulsions has been investigated by Olsson et al. [25,26], Dash et al. [27,28] and Piskunov et al. [29,30]. These investigations mainly focus on experimental analysis, which is not the mechanism that affects the factors of emulsion formation and stability. Numerical simulations provide a viable strategy to investigate the mechanism in order to overcome practical limitations. 

Computer simulations emerged as a powerful tool for studying the microstructures of amphiphilic copolymers [31]. Molecular dynamic simulation has been used more for studying the phase behavior [32,33,34]. Ma et al. adopted the molecular dynamic simulation to reveal the molecular mechanisms on the stability and instability of the interfacially active asphaltenes (IAA) stabilized O/W emulsions [30]. Dissipative particle dynamics (DPD) is frequently adopted for liquid systems and can reflect the dynamics on the mesoscopic molecular level of complex fluid systems, as one method of molecular dynamics simulation [35,36,37,38,39]. Furthermore, DPD can provide both the equilibrium thermodynamic properties and the dynamic details and the structural changes over time, which are either difficult or impossible to obtain by measurements [40,41,42]. Rekvig et al. successfully used a DPD simulation to investigate the influence of surfactant branching on the interfacial properties [43]. Wang et al. modified the DPD method, which is an excellent alternative to observe the interfacial properties of surfactant, oil and water systems at various temperatures and salts [44]. Accordingly, herein, the purpose of this study is to reveal the phase inversion and the stability factors of W/O and O/W emulsions using the DPD simulation method. We hope, according to this work, to observe a new way to form a more stable microemulsion and broaden the applications of microemulsions in many industries.

## 2. Simulation Methodology

### 2.1. Dissipative Particle Dynamics Theory

Dissipative particle dynamics (DPD) was firstly reported by Koelman and Hoogerbrugge as an efficient mesoscopic-level simulation method [36]. Several atoms or molecules are represented by beads that interact with each other via effective pair potentials. To simplify the calculations, the beads have the same mass, length, and time scales, in which the mass of the beads equals to 1 DPD unit. Every two beads *i* and *j* in a system interact with each other by the following formula from Groot [37]:

(1) $$f_{ij}=F_{ij}^{C}\left(r_{ij}\right)+F_{ij}^{R}\left(r_{ij}\right)+F_{ij}^{D}\left(r_{ij}\right)$$

where *F_ij_^C^*, *F_ij_^R^* and *F_ij_^D^* denote a conservative force, a random force and a dissipative force, respectively. In which, *F_ij_^C^* contains a harmonic spring force (*F_ij_^Cr^*) and a soft repulsion force (*F_ij_^Cs^*), which are given by

(2) $$\begin{array}{l} F_{ij}^{Cr}=\alpha_{ij}\left(1-\frac{r_{ij}}{r_{c}}\right)\frac{{\overset{\wedge }{r}}_{ij}}{r_{ij}}(r_{ij}<r_{c})\\ =0(r_{ij}>r_{c}) \end{array}$$

and

(3) $$F_{ij}^{C_{S}}=-C\cdot {\overset{\wedge }{r}}_{ij}$$

Among them, *α_ij_* is the maximum repulsion parameters between particles *i* and *j*, ${\overset{\wedge }{r}}_{ij}={\overset{\wedge }{r}}_{i}-{\overset{\wedge }{r}}_{j}$, *r*_*ij*_ = |${\overset{\wedge }{r}}_{ij}$|, *r_ij_* is the distance between *i* and *j*, with the corresponding unit vector ${\overset{\wedge }{r}}_{ij}$, *r_c_* is a cutoff radius which provides the extent of the interaction range and *C* is the spring constant. Moreover, the random force ($F_{ij}^{R}$) and the dissipative force ($F_{ij}^{D}$) can be shown by the following equations from Groot [37]:

(4) $$F_{ij}^{R}=\sigma \omega^{R}\left(r_{ij}\right)\theta_{ij}{\overset{\wedge }{r}}_{ij}$$

(5) $$F_{ij}^{D}=-\eta \omega^{D}\left(r_{ij}\right)\left(r_{ij}\cdot \nu_{ij}\right)\theta_{ij}{\overset{\wedge }{r}}_{ij}$$

Here, *θ_ij_* is the random fluctuation variable between 0 and 1, *v_ij_* represents the relative velocities of the beads, and *ω* is the weight function. Furthermore, *h* is the friction coefficient and *s* is the noise amplitude, and $\sigma^{2}=2\eta k_{B}T$. To sample the canonical ensemble distribution, *s*, *h* and *α_ij_* determine the amplitude of the dissipative, conservative and random forces [44].

$\omega^{D}={\left(\omega^{R}\right)}^{2}$ was made to comply with the fluctuation-dissipation theorem, and the temperature follows from the relation between *h* and *s*. The same parameters, weight functions, and integration algorithm were used from Groot and Warren [37]:

(6) $$\omega^{C}\left(r_{ij}\right)=\omega^{R}\left(r_{ij}\right)=\sqrt{\omega^{D}\left(r_{ij}\right)}=\omega \left(r_{ij}\right)$$

where

(7) $$\omega \left(r_{ij}\right)=\{\begin{array}{c} 1-\frac{r}{R_{C}}(r<R_{C})\\ 0\left(r\geq R_{C}\right) \end{array}$$

A modified version of the velocity verlet algorithm is adopted in the Newton’s equations of motion and the reduced units are used in our paper. Cutoff radius *R_c_*, k_B_*T* and m of the particles are used as the unit of length, energy and mass, respectively. Here, k_B_*T* represents the micro temperature, in which k_B_ is boltzmann constant and T the thermodynamic temperature. *h* = 4.5 and *s* = 3 are set in our research.

### 2.2. Models and Interaction Parameters

**Figure 1:**
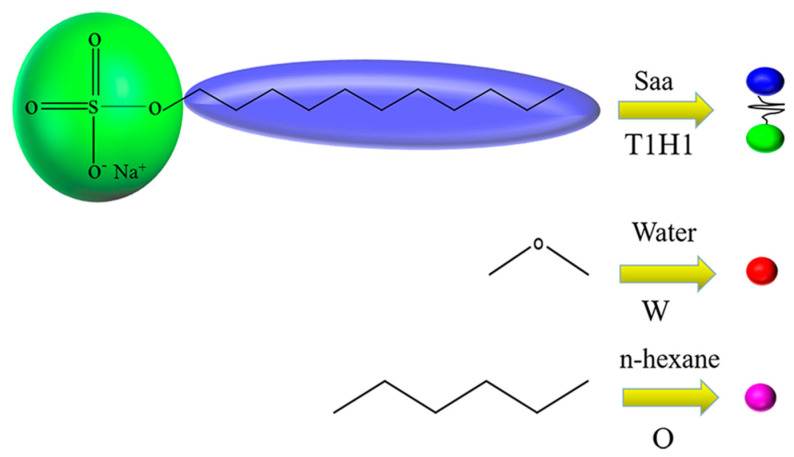
A coarse-grained model for surfactant, water and oil.

Water, oil of n-hexane and surfactant of sodium lauryl sulfate (SDS) were included in our research system. The coarse-grained models and shorthand notation for each molecular are presented in Figure 1. SDS are separated into two groups of hydrophilic and hydrophobic parts with the beads H set in green and T designated in blue, respectively. The H and T connected by a harmonic spring can be denoted by the symbol H1T1. Water is bead W in red, n-hexane is bead O in rose.

To clearly show the simulation results, the periodic boundary condition of three directions was employed in the cubic simulation box, which was 15 × 15 × 15 *R_c_*^3^ (L_x_ × L_y_ × L_z_). There were approximately 10,125 beads in every simulation box, and the density of beads was set to *ρ* = 3.0. It is possible to convert the simulation surfactant concentration to the mole fraction with the isochoric property. Based on Groot’s reports [37], the spring constant of every bead was set to 4.0.

**Figure 2:**
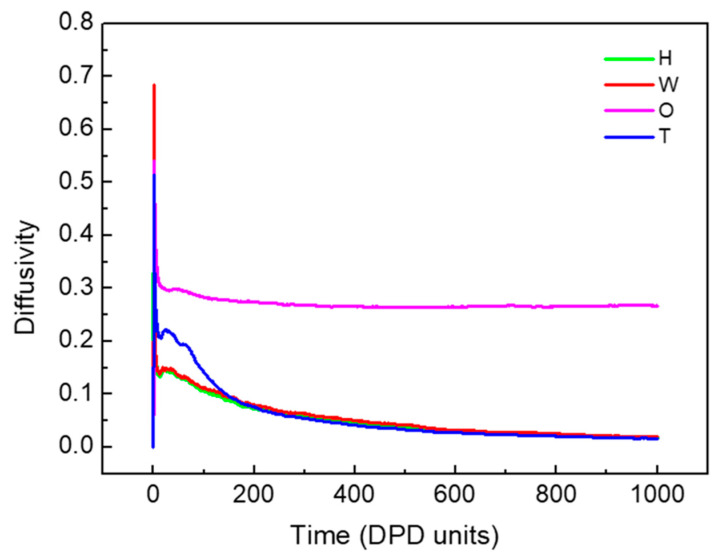
The diffusivity of beads H, T, W and O with the simulation time. The oil-water ratio was equal to 3:1.

The diffusivities of beads changed with an increasing simulation time, and are shown in Figure 2. A gradual decrease was observed with an increasing simulation time until 600 DPD units, and then remained unchanged after 800 DPD units. The simple modification was conducted following the velocity–varlet algorithm reported by Groot and Warren [37] and set ∆*t* = 0.05. Therefore, it is an equilibrium state can be reached by 20,000 timesteps simulations.

Table 1 is the repulsive interaction parameters between different beads referred to the previous reports [40,41,45].

## 3. Results and Discussion

### 3.1. Transformation of W/O and O/W Microemulsion Systems

#### 3.1.1. Dynamics of the W/O and O/W Microemulsion Systems Formation

**Figure 3:**
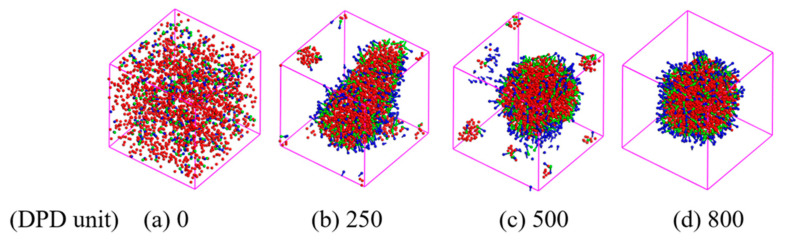
Snapshots of the evolution of the W/O microemulsion structure in DPD simulations with oil/water = 1/3. The hydrophilic group of surfactants are shown in green; the hydrophobic group is shown in blue. The water beads is shown in red. To clarity observe the internal structure, oil beads (pink) are not displayed.

A rigorous strategy for the formation of the W/O and O/W microemulsion systems is proposed, which provides the microstructure of formed microemulsions. We chose pure water and n-hexane as two incompatible systems, sodium lauryl sulfate as a surfactant to simulate the microemulsion by dissipative particle dynamics (DPD) simulation. The typical snapshots illustrating the evolution of the microemulsion structure with time as an example are presented in Figure 3, where the surfactant concentration is 0.1 and the value of oil-water is 1/3. To better observe the internal structure of the microemulsion, the oil beads were not exhibited in the simulation system. In the initial state (a), surfactants, oil and water were randomly dispersed in the simulation box. At a more extensive simulation time of 250 DPD units (b), the monolayer becomes gathered and the surfactant hydrophilic group associate with the water molecules to form a sheet aggregate. A further increase in the simulation time of 500 DPD units leads to the formation of the W/O microemulsion (c). Most surfactant molecules associate in the oil-water interfaces compactly and little surfactant molecules associate to form a little micelle in the system. Surprisingly, the little micelle disappears with increasing the simulation time to 800 DPD units (d), indicating that the interface of the microemulsion approaches the maximum interface concentration and forms a stable microemulsion. Therefore, the simulation time of 1000 DPD units is enough to create the microemulsion.

#### 3.1.2. Influence of Oil-Water Ratio on the Transformation of W/O and O/W Microemulsion Systems

**Figure 4:**
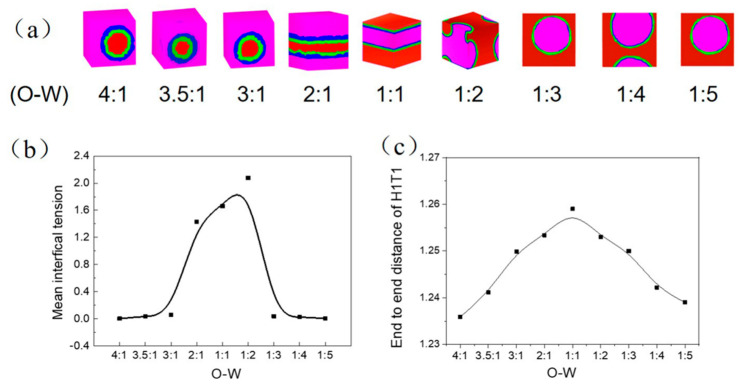
The translucent three-dimensional structure of the simulation system (**a**), the corresponding mean interfacial tension (**b**) and end to end distance of H1T1 (**c**).

The Oil-Water ratio could influence the emulsion stability and the emulsion formation of O/W or inverse W/O under high-energy input, which has extra stability with various affecting factors [23]. We set up the value of oil/water from 4:1 to 1:5 to study the microemulsion type affected by the oil/water ratio with the surfactant concentration of 0.05. Figure 4 shows the translucent three-dimensional structure of the simulation system (a), the corresponding mean interfacial tension (b) and end to end distance of H1T1 (c). The Irving and Kirkwood (IK) method was adopted to analyze the mean interfacial tension by:

(8) $$r_{sim}=\frac{1}{2}\int_{-L_{Z}/2}^{L_{Z}/2}[P_{N}\left(Z\right)-P_{L}\left(Z\right)]dz$$

Among them, *P_N_*(*Z*) was the pressure normal to the interface, the same as *P_zz_*(*Z*). The lateral force was given by *P_L_*(*Z*) = 1/2[*P_xx_*(*Z*) + *P_yy_*(*Z*)] with the pressure tensor component in the *Z* direction. The reality units could be transformed from the mean surface tension satisfied using the simulations by *γ* = *γ_sim_* × k_B_*T*/*R_c_*^2^, with *R_c_* = 0.711 nm and *T* = 298 K [39].

As the same content of oil and water as the oil/water = 1/1, surfactants adsorb at a flat interface and form a layer-like aggregate. The highest mean interfacial tension and end to end distance of H1T1 indicate that the surfactant molecular chain is most extended and has the weakest surfactant activity and emulsification capacity. With increasing oil content to the oil/water = 2/1, the increased oil phase with small amount was not sufficient to change the layer oil-water interface. The oil phase is sufficient to wrap the water phase and begin to form W/O microemulsion when the oil content increases to the oil/water = 3/1. Simultaneously, the decrease of the mean interfacial tension and end to end distance of H1T1 indicate the surfactant molecular chain owns a degree of bending and better emulsifying capacity. It is mainly due to the reduced oil and water interface with a larger oil phase and smaller water phase at the same surfactant concentration. As the oil phase increased to 3.5/1 and 4/1, the W/O microemulsion proved to be more stable, with a shrinking end to end distance of H1T1; however, the mean interfacial tension remained unchanged. Instead, O/W microemulsion was formed by increasing the water content to oil/water = 1:3. The mean interfacial tension reached its minimum value and did not change despite the addition of more water molecules. A gradual decrease in the end to end distance of H1T1 shows that the surfactant molecules were more compact and orderly at the interface and the O/W microemulsion is more stable.

**Figure 5:**
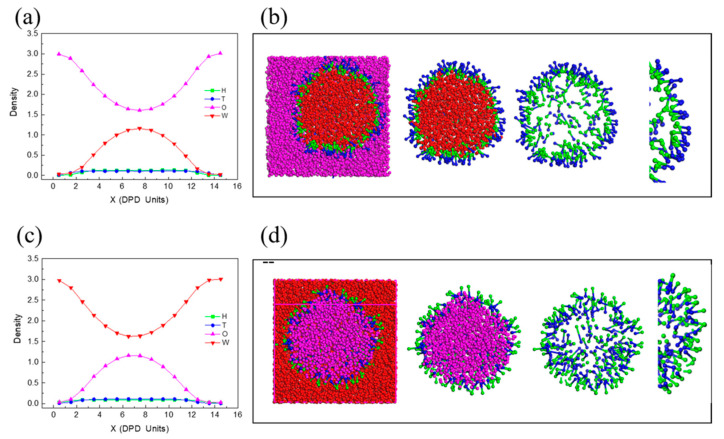
The density distribution of beads (H, T, W, O) along the *x*-axis in different oil-water ratios corresponding the translucent three-dimensional structures: (**a**,**b**) O/W = 3/1, (**c**,**d**) O/W = 1/3.

Figure 5 shows the density distributions of beads (H, T, W, O) along the *x*-axis in different oil-water ratios (3/1 and 1/3) corresponding the translucent three-dimensional structure used to observe the actual adsorption of surfactant molecules at the oil and water interface. W/O microemulsion is formed with the raised curve of W shown in Figure 5a, and the droplet size is around 12 DPD units (from 2 to 14 DPD units). Meanwhile, the internal water phase associated with the hydrophilic head makes the density of H slightly higher than T, which is caused by the smaller space of the internal water phase than the outside oil phase, with the same amounts of H and T. The translucent three-dimensional structure (Figure 5b) could observe this structure more intuitively. In contrast, the raised curve of O and T was slightly higher than H (Figure 5c) indicate the formation of O/W microemulsion. Figure 5d shows the inner structure of the formed O/W microemulsion, of which the hydrophobic group associated with the surface of the oil phase and the hydrophilic group disperse in the water phase.

### 3.2. Stability of the W/O and O/W Microemulsion Systems

#### 3.2.1. Influence of Temperature on the W/O and O/W Microemulsion Systems

**Figure 6:**
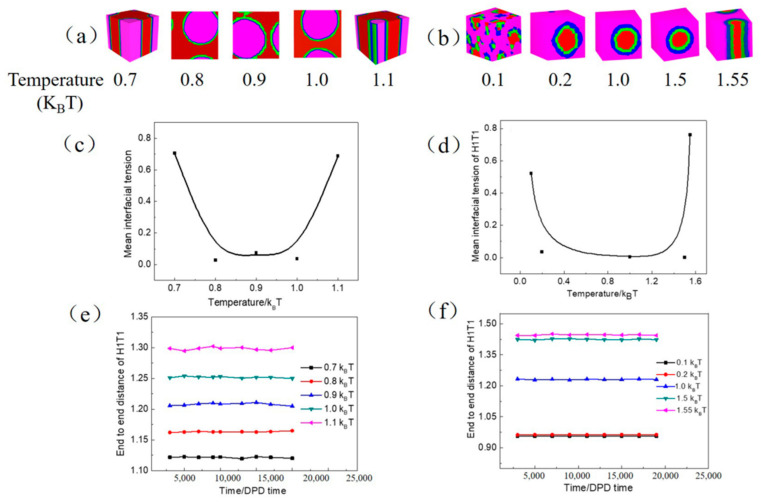
The translucent three-dimensional structure of microemulsion—(**a**,**b**), the corresponding mean interfacial tension—(**c**,**d**) and end to end distance of H1T1—(**e**,**f**) with increasing temperature. In which, (**a**,**c**,**e**) represent O/W microemulsion, (**b**,**d**,**f**) represent W/O microemulsion.

For safety, some food products based on emulsion often require heating treatment such as cooking and pasteurization. However, the emulsion could transform from W/O emulsion to O/W emulsion by changing the temperature from that observed in previous studies [18,24,44]. Therefore, the stability of the emulsion will be affected by the temperature. Figure 6 shows the impact of temperature on the W/O and O/W microemulsions. From the translucent three-dimensional structure of O/W microemulsion (Figure 6a), we can observe the minimum value for the stability of O/W microemulsion at 0.8 k_B_*T*, but the maximum value at 1.0 k_B_*T* with the minimal mean interfacial tension (Figure 6c). However, a greater increase is observed in the mean interfacial tension after heating over 1.0 k_B_*T* or cooling to 0.8 k_B_*T*. This may be due to the O/W microemulsion transformed into a rod structure with a more extensive oil-water interface than microemulsion, which reduces the ability to change the surface activity of H1T1. End to end distance of H1T1 (Figure 6e) gradually increases with increasing the temperature caused by more extension molecular chain consistent with the results obtained by Chen et al. [46].

By contrast, the W/O microemulsion demonstrated a better stability resistance to temperature as long as the temperature is below 1.55 k_B_*T*, as shown in Figure 6b. However, one big W/O droplet was separated into a number of tiny droplets when the temperature decreased to 0.1 k_B_*T*. Compared with O/W microemulsion, the better stability of W/O microemulsion may be due to the higher viscosity of oil molecules that were not easily spread in the decentralized system.

#### 3.2.2. Influence of Inorganic Salt on the W/O and O/W Microemulsion Systems

**Figure 7:**
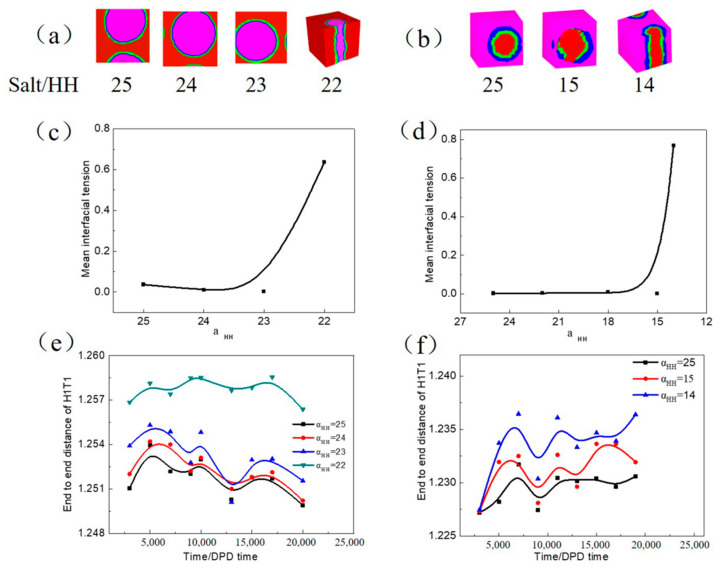
The translucent three-dimensional structure of microemulsion—(**a**,**b**), the corresponding mean interfacial tension—(**c**,**d**) and end to end distance of H1T1—(**e**,**f**) with increasing inorganic salt. In which, (**a**,**c**,**e**) represent O/W microemulsion, (**b**,**d**,**f**) represent W/O microemulsion.

Two salts (NaCl and CaCl_2_) affect the emulsion stability and were observed using experiment and simulation by Zhong et al. [20] and Zhang et al. [47]. Despite this, we investigated the influence of salt on the stability of W/O and O/W microemulsion by the DPD simulation shown in Figure 7. The decrease in head–head repulsion parameters (*α_HH_*) means adding the inorganic salt, and if no inorganic salt exists *α_HH_* = 25. The translucent three-dimensional structure of O/W microemulsion (Figure 7a) exhibits that the droplet transform into the rod topology with decreasing *α_HH_* to 22; meanwhile, a significant increase occurred in the mean interfacial tension (Figure 7c). There are certain fluctuations in end to end distance of H1T1 with increasing simulation time whether the inorganic salt is added shown in Figure 7e. This is mainly due to the different degree of adsorption of surfactant molecules at the oil-water interface. At the beginning of the simulation, the surfactant molecules were scattered in an aqueous solution and showed a certain degree of bending because of the intermolecular repulsion. With the increase in simulation time, small unstable microemulsion droplets gradually formed and finally reached the equilibrium state to form one stable and large microemulsion droplet and resulted in different degrees of bending. However, end to end distance of H1T1 gradually increases with increasing salt concentration (Figure 7e). It was probably caused by the opposite ion of inorganic salt could neutralize part of the charge of the hydrophilic group and reduce the electrostatic repulsion between the hydrophilic groups. It leads to looser surfactant molecules at the oil and water interface and the molecular chains are more extended.

However, W/O microemulsion begins to transform when *α_HH_* decreases to 14 (Figure 7b). The rod topology has the highest interface tension (Figure 7d) and most significant end-to-end distance (Figure 7f). W/O microemulsion has a better stability resistance to the inorganic salt compared with O/W microemulsion, which is consistent with the results observed by Huang et al. [24]. This may be due to the adsorption position of H1T1 at the oil and water interfaces. The hydrophilic group associated with the interface caused a more significant spatial block effect and hindered the electrostatic gravity of inorganic salt and hydrophilic ions in W/O microemulsion.

#### 3.2.3. Influence of Shear on the W/O and O/W Microemulsion Systems

**Figure 8:**
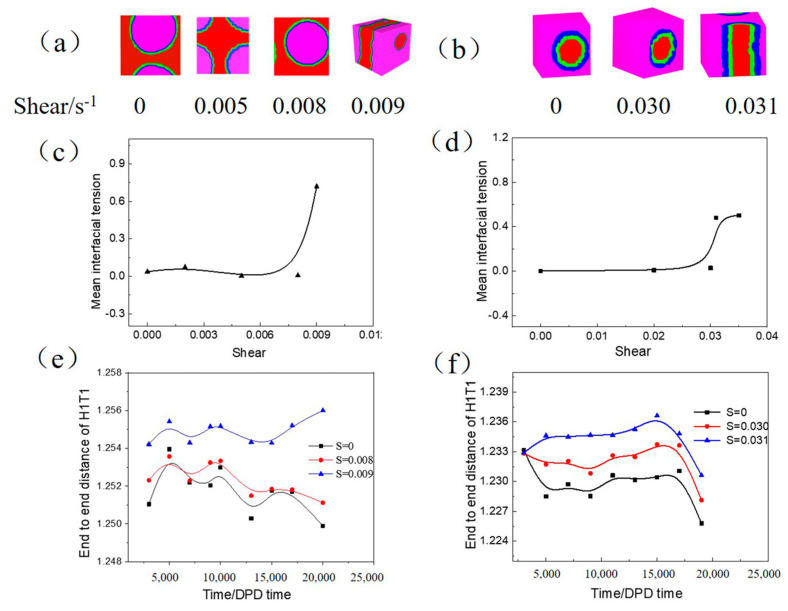
The translucent three-dimensional structure of microemulsion—(**a**,**b**), the corresponding mean interfacial tension—(**c**,**d**) and end to end distance of H1T1—(**e**,**f**) with increasing shear rate. In which, (**a**,**c**,**e**) represent O/W microemulsion, (**b**,**d**,**f**) represent W/O microemulsion.

Lee–Edwards sliding-brick boundary conditions along *x*-axis were applied to the simulation system representing the impact of shear flow on the structure and orientation of complex fluids. Figure 8 reveals the influence of shear on the O/W and W/O microemulsion. The topology of the O/W microemulsion droplet is stable in the absence of shear and maintains this structure when increasing the shear rate to 0.008s^−1^. However, the oil cannot be wrapped by water after the shear rate is enhanced to 0.009s^−1^ and transformed to a layer-like aggregate (Figure 8a) with a significant increase in the mean interfacial tension (Figure 8c). This is mainly due to the shear rate inducing the aggregate spread out along the direction of the shear rate, similarly to an external force to the microemulsion. An increase in the end to end distance of H1T1 for O/W microemulsion indicates that the molecular chain is more extended with a larger shear rate (Figure 8e). However, for W/O microemulsion, the droplet emerges deformation until the shear rate to 0.031s^−1^ (Figure 8b) with a more considerable mean interfacial tension (Figure 8d) and end to end distance of H1T1 (Figure 8f). In light of this, W/O microemulsion has better stability to resist the shear rate owing to the higher viscosity of the oil phase as the continuous phase is not easy to affect by an external force.

## 4. Conclusions

The topology and stability of O/W and inverse W/O microemulsion were studied using the dissipative particle-dynamics simulation method. Coarse-grained models were constructed for surfactant (H1T1), oil and water, respectively. The results show that the ratio of oil and water would change the topology of the microemulsion that transforms from W/O to O/W by decreasing the value of oil/water from 3:1 to 1:3. Meanwhile, the effects of the temperature, inorganic salt and shear rate on the stability of the formed microemulsions were researched with a translucent three-dimensional structure and corresponding parameters such as mean interfacial tension and end-to-end distance of H1T1. Inverse W/O microemulsion has better resistance to a higher temperature (1.5 k_B_*T*), inorganic salt (*α_HH_* = 14) and shear rate (0.03 s^−1^) than the O/W microemulsion of *T* = 1.0 k_B_*T*, *α_HH_* = 23 and s = 0.008 s^−1^. In the inverse W/O microemulsion of oil as the continuous phase with higher viscosity is not easy to affect the physical and chemical properties. The simulation provides a powerful tool to forecast the structure and the stability of various microemulsions, which is of great importance for developing new functional emulsions for many applications.

## Figures and Tables

**Table 1:** The interaction parameters employed in this simulation.

	W	H	T	O
W	25			
H	25.34	25		
T	151.52	177.82	25	
O	103.24	143.61	25.94	25

## Sample Availability

Samples of all the compounds are available from the authors.
